# Stochasticity and non-additivity expose hidden evolutionary pathways to cooperation

**DOI:** 10.1371/journal.pone.0225517

**Published:** 2019-12-02

**Authors:** Sarah E. Fumagalli, Sean H. Rice

**Affiliations:** Department of Biological Sciences, Texas Tech University, Lubbock, Texas, United States of America; University of Sheffield, UNITED KINGDOM

## Abstract

Cooperation is widespread across the tree of life, with examples ranging from vertebrates to lichens to multispecies biofilms. The initial evolution of such cooperation is likely to involve interactions that produce non-additive fitness effects among small groups of individuals in local populations. However, most models for the evolution of cooperation have focused on genealogically related individuals, assume that the factors influencing individual fitness are deterministic, that populations are very large, and that the benefits of cooperation increase linearly with the number of cooperative interactions. Here we show that stochasticity and non-additive interactions can facilitate the evolution of cooperation in small local groups. We derive a generalized model for the evolution of cooperation and show that if cooperation reduces the variance in individual fitness (separate from its effect on average fitness), this can aid in the evolution of cooperation through directional stochastic effects. In addition, we show that the potential for the evolution of cooperation is influenced by non-additivity in benefits with cooperation being more likely to evolve when the marginal benefit of a cooperative act increases with the number of such acts. Our model compliments traditional cooperation models (kin selection, reciprocal cooperation, green beard effect, etc.) and applies to a broad range of cooperative interactions seen in nature.

## Introduction

Cooperation in which individuals interact and generate fitness benefits either at the individual or group level is found in an immense array of biological systems. Of particular interest is the evolution of costly cooperation. Throughout this discussion, we define a costly cooperative trait as one that reduces the total expected relative fitness of the individual that expresses it, all else held equal, while increasing the total expected relative fitness of another. In this analysis, we will illustrate some ways in which stochastic fitness and non-additive benefits facilitate the evolution of cooperation.

Historically, most models for the evolution of cooperation have focused on genealogically related individuals [1–7]; assume that the factors influencing individual fitness are deterministic [5,6, 8–15,16–22], that populations are very large [1–4,13,15–17,23–26], and that the individual benefits of cooperation are additive [1,2,5,6,12–17,20–22,26–31]. A number of authors have attempted to relax some of these assumptions by including individual stochasticity [7,23,32–38] and non-additivity [7,23,32–35]. However, none of these studies relax all of the assumptions aforementioned at the same time, while considering the impacts of variance at both the individual and population levels.

**Fig 1:**
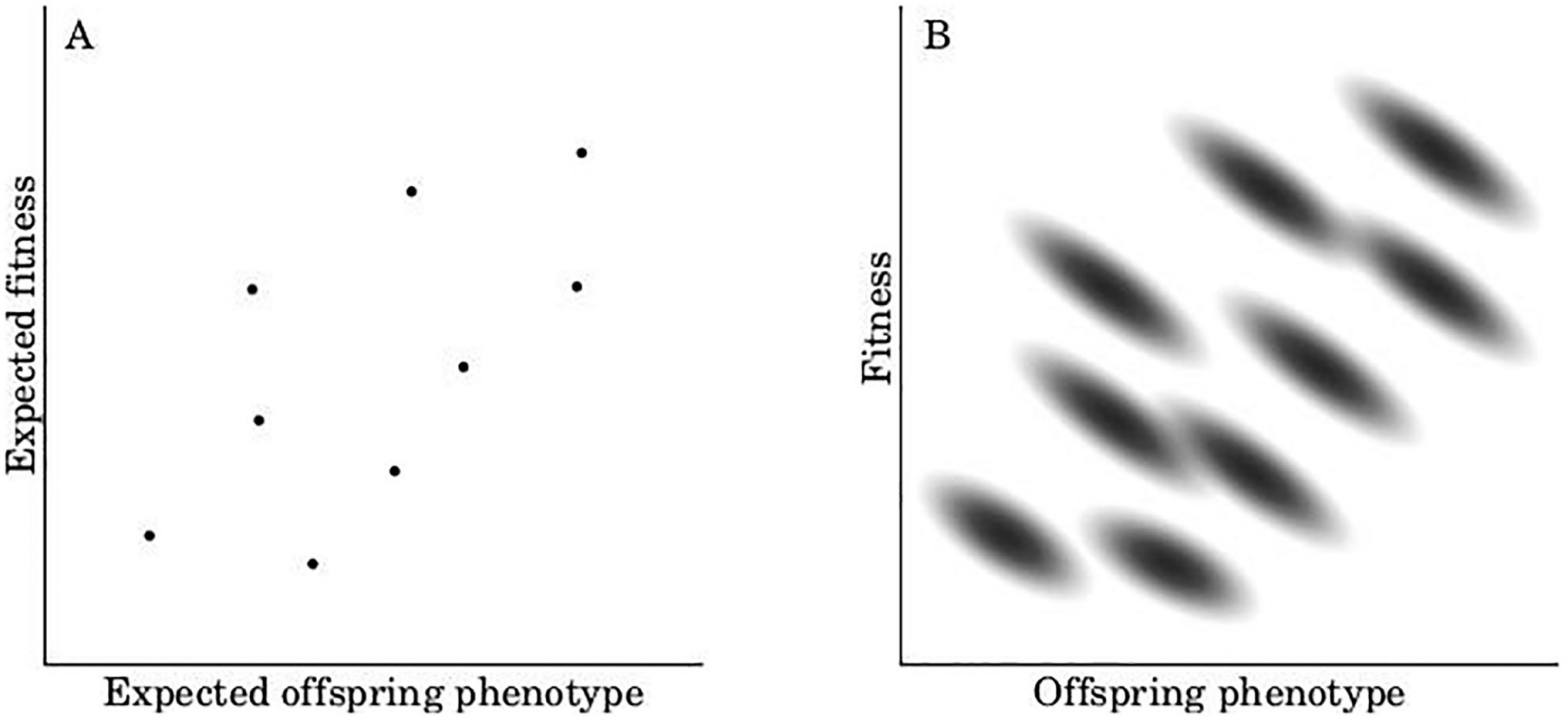
Distinguishing between frequency and probability operations. Illustration of the difference between frequency and probability operations. (A) shows the frequency distribution, for a hypothetical population, of expected offspring phenotype and expected absolute fitness. Since these are expected values, each individual corresponds to a point. In (B), we plot offspring phenotype verses fitness–not their expectations. In this case, each individual corresponds to a probability distribution of values.

In natural populations, there is a sizeable stochastic component to individual fitness [23, 39–54]. This means that we cannot say with certainty the number of descendants any given individual will leave. We thus need to treat fitness as a random variable–each individual having a probability distribution of possible fitness values (Fig 1B). These probability distributions must be distinguished from the frequency distribution of variation within a population. We thus need to distinguish two different kinds of statistical operations in our models (Fig 1). We will use a bar (e.g. $\overset{-}{w}$) to denote the frequency mean across a population, and hat (e.g. $\hat{w}$) to denote the expected value of a random variable that has a probability distribution (this follows the notation in Rice and Papadopoulos 2009; note that a hat denotes an expected value, not an estimator, of a random variable [55]).

Fig 1A shows the joint distribution of expected average offspring phenotype ($\hat{\phi_{i}^{o}}$) and expected absolute fitness (${\hat{w}}_{i}$) for a hypothetical population. There is variation between individuals across the population, but zero variation within individuals (intra-individual variation). In this case, there is a positive frequency covariance (denoted $⟦\hat{\phi_{i}^{o}},{\hat{w}}_{i}⟧$) between these variables (Table 1). We use the term ‘frequency covariance’ because it is calculated across a frequency distribution of individuals.

**Table 1:** Term and symbol definitions for the non-additive, deterministic case.

*B*	number of offspring produced with help
***C***	difference between non-cooperators’ and cooperators’ baseline fitness distribution; a cooperator’s cost for helping
***w*_*i*_**	absolute fitness for individual *i*
***w*_0_**	number of offspring produced without help; baseline fitness
***ρ*_*i*_**	number of cooperators individual *i* interacts
***P***	the degree to which cooperation is directed at cooperators verses non-cooperators
$\phi_{i}^{o}$	mean offspring phenotype for individual *i*
***δ***	differences between offspring and parental phenotype due to processes other than selection (i.e. recombination, transmission)
**⟦^2^*x*⟧**	frequency variance in *x* across the population
**⟦*x*, *y*⟧**	frequency covariance between *x* and *y* across the population
$\overset{-}{x}$	mean of *x* values for a population

In reality, neither offspring phenotype nor fitness can be predicted with certainty prior to reproduction. Fig 1B shows a hypothetical plot of these variables by themselves–not their expected values. Each individual in the current generation now has a distribution of $\phi_{i}^{o}$ and *w*_*i*_. In the example shown, there is a negative probability covariance (denoted $\left\langle \left\langle \phi_{i}^{o},w_{i}\right\rangle \right\rangle$) for each individual (Table 2). This means that if an individual happens to leave more offspring than expected (its actual fitness, *w*_*i*_, turn out to be higher than its expected fitness, ${\hat{w}}_{i}$), then its offspring will likely be smaller than they would be if there were fewer of them. We call these ‘probability covariances’ because they are calculated across a probability distribution of possible future outcomes. In general, there is no reason to expect the frequency covariance across a population to be the same as the probability covariance for any one individual.

**Table 2:** Term and symbol definitions for the non-additive, stochastic case.

*B*	probability distribution of possible numbers of offspring produced with help
***B*_0_**	probability distribution of possible linear fitness benefits
***β***	probability distribution of possible non-linear fitness benefits
***C***	probability distribution of possible differences between non-cooperators’ and cooperators’ baseline fitness distribution; cost to cooperator for helping
***w*_*i*_**	probability distribution of possible absolute fitness values for an individual
***w*_0_**	probability distribution of possible numbers of offspring produced without help; baseline absolute fitness
***ρ*_*i*_**	probability that individual *i* interacts with an cooperator
***P***	probability distribution of possible differences in the degree to which cooperation is directed at cooperators verses non-cooperators
$\phi_{i}^{o}$	probability distribution of possible mean offspring phenotypes of individual *i*
***δ***	probability distribution of possible differences between offspring and parental phenotype due to processes other than selection (i.e. recombination, transmission)
***Ω*_*i*_**	probability distribution of possible relative fitness values of individual *i*
$\hat{x}$ **or *E(x)***	expectation or expected value of random variable *x*
$\overset{-}{x}$	probability distribution of possible mean of *x* values for a population
**≪*x*, *y*≫**	probability covariance between random variable *x* and random variable *y* of individual *i*
**≪^2^*x*≫**	probability variance in random variable *x* of individual *i*
$H(\overset{-}{w})$	probability distribution of possible values of harmonic mean of mean population fitness
$\hat{\overset{-}{\Delta A}}$	expected average change in cooperation over time

To create a generalized model of the evolution of cooperation, we assume populations are finite, that individuals can interact in any size groups, and that the components of individual fitness–including the costs and benefits of cooperation–are random variables, here captured with probability distributions (See Table 2 for definitions). Since we are adding stochasticity to models that already involve operations like covariances and regressions, we have to be clear about our notation. The term ‘random variable’ is sometimes used to describe any value for which we can calculate a mean, variance, or a covariance. We are using the term in this way. We will use *random variable* to refer to a variable that has a probability distribution of possible states, due to the fact that it is influenced by one or more random processes–such as mutation, recombination, or unpredictable environmental variation. Under this definition, terms in the deterministic Price equation are not treated as random variables [56] (Table 1):

$$\Delta \overset{-}{\phi }=⟦\phi_{i}^{o},\Omega_{i}⟧+\overset{-}{\delta }$$ (1)

The covariance in Eq 1 is a frequency covariance, calculated over all individuals in a population. If we are treating the change in mean phenotype ($\Delta \overset{-}{\phi }$) as a determinate value, then we are tacitly assuming that each individual has a single value of $\phi_{i}^{o}$ and *Ω*_*i*_. This means that in Eq 1, $\phi_{i}^{o}$ and *Ω*_*i*_ are not strictly random variables–since they are not the result of any random process. Of course, if we were calculating the covariance of values in a random sample from a larger population (which is what statisticians are most often concerned with), then these terms would be random variables; inheriting their randomness from the sampling process. The Price equation, however, is concerned with the entire population, not a sample from it. We are thus, (in the case of Eq 1) dealing with a covariance between deterministic values.

This distinction becomes important when we want to introduce true stochasticity into our analysis. Now, $\phi_{i}^{o}$ and *Ω*_*i*_ for a particular individual become true random variables—meaning that there can be a probability covariance between them. When $\phi_{i}^{o}$ and *w*_*i*_ become random variables, the change in mean phenotype ($\Delta \overset{-}{\phi }$) and mean absolute population fitness ($\overset{-}{w}$) also become random variables, having a distribution of possible values. If we calculate the expected value of change in mean phenotype, we get the stochastic Price equation [57]:

$$\hat{\Delta \overset{-}{\phi }}=⟦\hat{\phi_{i}^{o}},\hat{\Omega_{i}}⟧+{\overset{-}{⟪\phi_{i}^{o},\Omega_{i}⟫}}_{i}+\hat{\overset{-}{\delta }}.$$ (2)

The first term on the right side of Eq 2 ($⟦\hat{\phi_{i}^{o}},\hat{\Omega_{i}}⟧$) is the same as the covariance in Eq 1, except that it explicitly involves the expected values of $\phi_{i}^{o}$ and *Ω*_*i*_. This is analogous to the covariance between the points in Fig 1A.

The second term in Eq 2 (${\overset{-}{⟪\phi_{i}^{o},\Omega_{i}⟫}}_{i}$) is the frequency average (i.e. the average across all individuals in the population) of the probability covariance between $\phi_{i}^{o}$ and *Ω*_*i*_ for each individual. Each individual has a joint distribution of possible mean offspring phenotypes and relative fitness values that have not been realized. This is like the average of the covariances for the different probability distributions in Fig 1B.

Eq 2 shows that introducing stochasticity can alter the expected change in mean phenotype. Below, we will show that the combination of stochasticity and non-additivity, reveals new mechanisms that can facilitate or impede the evolution of cooperation between genealogically unrelated individuals.

## Results

### The non-additive, deterministic case

Costly cooperation entails a fitness cost to the cooperator, *C*, and confers a fitness benefit, *B*, on recipients (whether they are cooperators or non-cooperators). For the rest of this discussion, we impose the cost as an inherent deduction from the maximal baseline fitness of cooperators, whether they interact with another individual or not. We are thus focusing on traits like overproduction of a metabolic compound, which incurs a cost to the cooperator regardless of whether there is another individual nearby to benefit from it. This is likely a common case in systems such as biofilms or slime molds. To capture any potential group structure within the population, we denote the amount of cooperation experienced by a cooperator as *ρ*_*A*_ and the amount experienced by a non-cooperator as *ρ*_*S*_ [9,26,29,58] (Table 1). *ρ*_*i*_ will be a function of the distribution of individuals within a population (See Methods –Simulation 1 and 2).

We will be focusing specifically on costly cooperation; meaning that we need to distinguish costs and benefits. If we were instead modeling mutualism, then the fitness components of interest may change (e.g. the cost term may be unnecessary) or the benefits of cooperation may entail details that are not necessarily captured. Using the notation in Table 1, we can now write the deterministic absolute fitness equations for each phenotype, cooperators and non-cooperators respectively:

$$w_{A}=w_{0}+B\rho_{A}-C;$$ (3)

$$w_{S}=w_{0}+B\rho_{S}.$$ (4)

For the moment, we are treating *w*_0_, *B*, *ρ*_*i*_, and *C* as deterministic (we relax this assumption in the next section). In the majority of cooperation models, the benefits of cooperation are assumed to be additive, meaning that the total fitness benefit from multiple cooperative interactions is the sum of the effects of each independent interaction. In fact, as with many components of fitness, the benefits of cooperation are likely to be non-additive [13,19,23,39,42–44,47–49,50–53,58,59]. Non-additivity means that the benefits an individual receives will change (may increase or decrease) as they interact with more cooperators. Non-additivity is often represented in terms of ‘synergistic’ benefits, though this ‘deviation from additivity’ produces the same benefit value each cooperator/cooperator interaction (e.g. Table 2 in [15]) [15–18,22]. By contrast, we will allow the benefits of cooperation to be a non-additive function of the number of cooperative interactions experienced, regardless of whether the recipient is an cooperator or not. This means that as the number of cooperative interactions increase for an individual over time, the benefit per interaction is greater or smaller than the benefit gained in the previous interaction.

To capture any possible non-additive benefit, we write the total benefit, *B*, as a function of the amount of cooperation experienced, *ρ*_*i*_ and a parameter ‘*β*’ (which determines the curvature of the function):

$$B=\beta \rho_{i}+\cdots .$$ (5)

**Fig 2:**
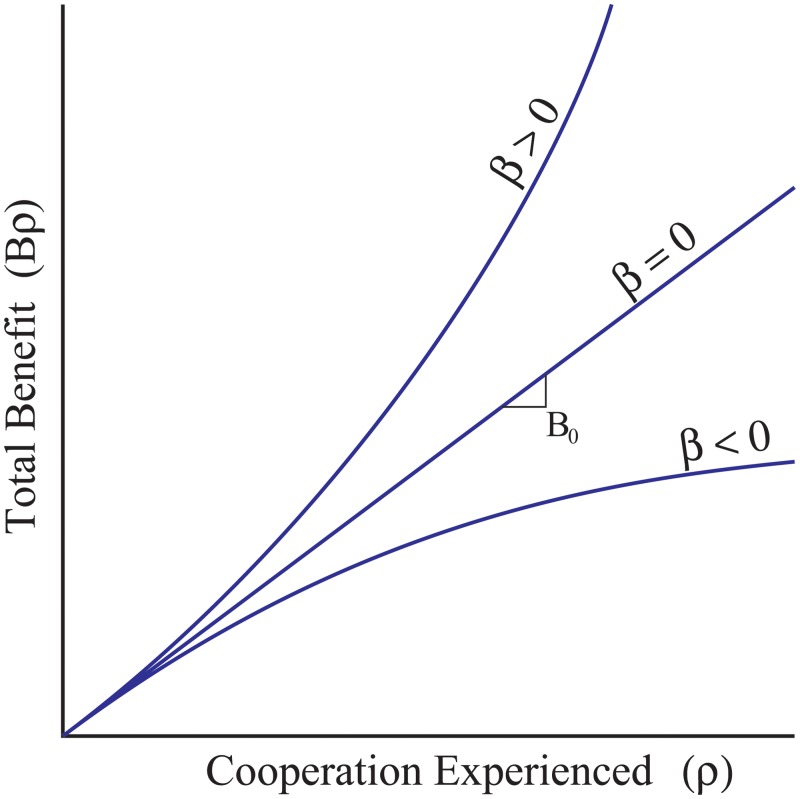
Possible additive and non-additive benefit functions. Three possible total benefit function examples that individuals may experience. If the total benefit of cooperation remains consistent as cooperation experienced increases, then benefits accrue additively (*B*_0_ ≠ 0, *β* = 0). If the total benefit accelerates (*B*_0_ = 0, *β* > 0) or diminishes (*B*_0_ = 0, *β* < 0) as cooperation experienced increases, then benefits accrue non-additively.

Fig 2 shows examples of how total benefit (*B*) can be related to *ρ*_*i*_ according to Eq 5. Fig 2 only shows three possible benefit functions, two of which are non-additive. This is not to say that these are the only possible benefit functions, other examples have been used [35]. In Eq 5 and Fig 2, setting *β* = 0 yields the additive case, in which the fitness benefit increases additively with experienced cooperation. Setting *β* > 0 means that the marginal benefit of a cooperative act increases with the number of such acts experienced. Conversely, *β* < 0 corresponds to cases in which there are diminishing returns from increasing experienced cooperation.

The cost of cooperation (*C*) could also be expanded in this way; if there is variation in how much cost is paid as the number of cooperative interactions increases. As noted above, however, we are considering cases in which the cost is an inherent property of being a cooperator. We thus will treat *C* as fixed. The standard condition of when to expect cooperation to increase under a deterministic model is when the average absolute fitness of cooperators is greater than the average absolute fitness of non-cooperators [5]:

$${\overset{-}{w}}_{A}>{\overset{-}{w}}_{S}.$$ (6)

By combining Eqs 3–6 and assuming *B*_0_ and *β* are constant across the population (all individuals have the same value; therefore, the average across them is also the same), we find the condition for cooperation to increase as a function of the amount of cooperation experienced by cooperators and non-cooperators (see Methods):

$$B_{0}P_{1}+\beta P_{v}+\beta P_{2}-C>0.$$ (7)

The deterministic ‘*P*’ terms in Eq 7 are functions of the (frequency) distributions of *ρ*_*A*_ and *ρ*_*S*_. They are defined as follows:

- $P_{1}\equiv ({\overset{-}{\rho }}_{A}-{\overset{-}{\rho }}_{S})$ → The average degree to which cooperation is directed towards cooperators minus the degree to which cooperation is directed at non-cooperators. The subscript 1 refers to a ‘first order genealogical relatedness-like’ term. This, like the following ‘*P*’ terms, may well change from generation to generation.
- *P*_*v*_ ≡ ((⟦^2^*ρ*_*A*_⟧ − (⟦^2^*ρ*_*S*_⟧) → The difference in frequency variance in cooperation experienced by cooperators and cooperation experienced by non-cooperators. The subscript *v* refers to a ‘variance capturing’ term. These frequency variances capture the variation across the population in cooperation experienced across cooperators (⟦^2^*ρ*_*A*_⟧) and non-cooperators (⟦^2^*ρ*_*S*_⟧).
- $P_{2}\equiv ({\overset{-}{\rho_{A}}}^{2}-{\overset{-}{\rho_{S}}}^{2})$ → The average degree to which cooperation is directed towards cooperators squared minus the degree to which cooperators is directed at non-cooperators squared. The subscript 2 refers to a ‘second order genealogical relatedness-like’ term.

Eq 7 shows that *P*_*v*_ and *P*_2_ become important when the benefits of cooperation are non-additive (*β* ≠ 0). *P*_1_ is similar to the “genealogical relatedness” term in kin selection models (though it allows interactions between non-relatives) [9,26,29,58]. Note that we are separating the degree of cooperation that one experiences (which goes into the *ρ*_*i*_ terms) from the fitness benefits that accrue from that cooperation (which do not influence the *ρ*_*i*_ terms). We are thus distinguishing between the act (such as the degree to which an individual bacterial cell overproduces a metabolic compound that might benefit those around it) and the consequences of that act for others (e.g. how much other cells actually benefit from that compound).

Some authors define relatedness in such a way that the fitness effects of an cooperative act are combined into the degree of cooperation expressed by an individual (or the genic value of an individual) [3,7,8,17,18,22]. In such models, any non-linearities in the benefits of cooperation (i.e. if *β* ≠ 0) are folded into relatedness. In our model, we distinguish the act from its fitness consequences both because they are biologically distinct (for example, an environmental change might influence one but not the other) and because they can potentially vary independently of one another. Note that under this definition, *P*_1_ may be zero (i.e. cooperators no more likely to interact with other cooperators than with non-cooperators), even when ″*r*″ ≠ 0 in models under which fitness effects are folded into the definitions of genic values [3,7,8,17,18,22]. Furthermore, when we make the model stochastic, we will show that individual behavior, fitness effects and clustering of individuals can have very different stochastic behavior. Studying interactions between these effects will reveal hidden processes influencing the evolution of cooperation.

Any behavior that increases *P*_1_, all else held equal, will increase the potential for cooperation to spread. Since the ‘*P’* terms simply measure the degree in individual cooperative interaction, this model accommodates different biological processes influencing why individuals encounter who they encounter. If all interactions are between genealogically unrelated individuals, *P*_1_ may be non-zero.

A number of proposed biological processes can increase *P*_1_, though they are usually treated in separate models. These include but are not limited to:

- Genealogical relatedness: One way to boost *P*_1_ in favor of cooperators is to direct their helping towards genealogical relatives. This case essentially captures kin selection, though note that we are using absolute fitness (‘neighbor modulated fitness’ in [5]) instead of inclusive fitness. Inclusive fitness models frame ‘relatedness’ in terms of the actor’s genetic value and the recipient’s genetic value. What matters in this model is that cooperative interactions are preferentially directed towards individuals that share alleles identical by descent [5]. *P*_1_ captures the relationship between the focal individual’s phenotype and how much cooperation they experience. It is thus not defined in terms of genealogical relatedness, but will tend to be larger if cooperators direct their helping preferentially towards relatives. In a very large population in which cooperators are very rare, *P*_1_ can often be approximated by genealogical relatedness.

**Fig 3:**
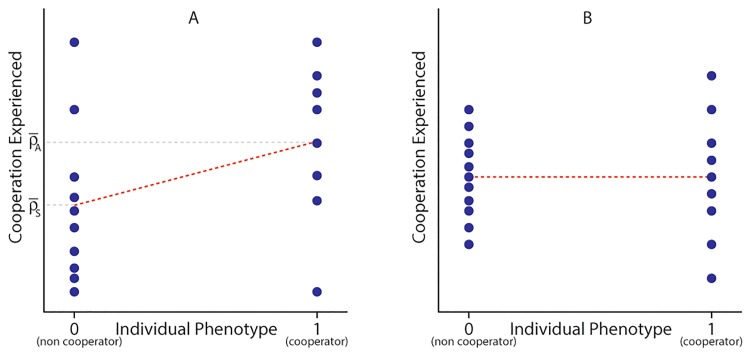
Cooperation experienced distributions for both phenotypes. Each dot represents the expected cooperation experienced for each individual according to their phenotype. The difference in the average of these distributions (${\overset{-}{\rho }}_{A}-{\overset{-}{\rho }}_{S}$) is equal to the regression of cooperation experienced on cooperation expressed. In case (A), cooperators interact with other cooperators more often than non-cooperators on average (*P*_1_ > 0). In case (B), cooperators interact with other cooperators just as often as non-cooperators on average (*P*_1_ = 0); therefore, the slope of the regression is zero, though the variance in cooperation experienced is not zero (*P*_*v*_ > 0).

*P*_1_ likewise encompasses more recent “statistical” definitions of relatedness (though not those that fold the fitness effects of cooperation into the measure of cooperation experienced). These define ‘relatedness’ as the linear regression of group composition (or cooperation experienced) on individual genotype or phenotype [8,11,14–17,23,30,31]. As Fig 3 shows, for a binary case with two states (cooperator and non-cooperator), (${\overset{-}{\rho }}_{A}-{\overset{-}{\rho }}_{S}$) is equal to the regression of cooperation experienced on cooperation expressed. If *P*_1_ < 0, then on average cooperators interact with other cooperators less often than with non-cooperators. In terms of the regression on actor and recipient genetic value, we are explicitly separating genetic value from the phenotype cooperation experienced, though, the non-additive benefits of cooperation will influence the difference in the variance of cooperation experienced (*P*_*v*_) and the ‘second order relatedness-like’ term (*P*_2_) [36,60].

- Past behavior as an indicator: Another way to increase *P*_1_ is for cooperators to direct help preferentially towards others who have themselves acted cooperative in the past (rather than due to genealogical relatedness). This phenomenon is generally folded into models of reciprocal cooperation, but the process we are highlighting here is different from the expectation of a future benefit [8,10,14,15,22–24,33,61]. In fact, the expectation of receiving a returned benefit is not necessarily non-zero.
- Recognizable cooperative trait: Here, cooperators direct cooperation towards others who express some indicator trait (e.g. ‘green beard’) that correlates with the recipient’s potential cooperative behavior. Theoretical models and empirical experimentation have shown that such indicator traits can facilitate the evolution of cooperation, even among microbial colonies [2,8,62–65].

These are three well-known ways to increase the likelihood of an cooperative interaction, and thus increase *P*_1_. Though, we cannot say that simply because *P*_1_ is positive that cooperation will increase. For example, if cooperators have lower variance in cooperation experienced than non-cooperators (i.e. most cooperators experience similar amounts of cooperation, while some non-cooperators have many cooperative experiences, most do not), then *P*_*v*_ will be negative. If, in addition, *β* > 0, then this negative *P*_*v*_ will reduce the potential for cooperation to increase. Decreasing benefits (*β* < 0) will facilitate the increase in cooperation if *P*_*v*_ is also negative.

As noted above, our model distinguishes the amount of cooperation experienced from the fitness benefits that result from it. How the magnitude of the benefits of cooperation changes plays a big role in influencing the evolutionary outcome. If *β* = 0 in Eq 5 (meaning that fitness contributions are additive), then Eq 6 is just a simple version of Hamilton’s rule, equivalent to results in many other studies [1,4,5,6,12–17,20–22,26,28–31]. When the effects of cooperation are explicitly non-additive, interestingly two new terms appear in Eq 7, *β*((⟦^2^*ρ*_*A*_⟧ − ⟦^2^*ρ*_*S*_⟧) and $\beta ({\overset{-}{\rho_{A}}}^{2}-{\overset{-}{\rho_{S}}}^{2})$. The term $\left({\overset{-}{\rho_{A}}}^{2}-{\overset{-}{\rho_{S}}}^{2}\right)$ (‘second-order relatedness’) is always of the same sign as the first-order relatedness-like term, $\left({\overset{-}{\rho }}_{A}-{\overset{-}{\rho }}_{S}\right)$, but is multiplied by *β*, which measures non-additivity of benefits (Fig 2). Thus, compounding fitness effects (*β* > 0) increase the potential for cooperation to spread when $({\overset{-}{\rho }}_{A}-{\overset{-}{\rho }}_{S})>0$. Conversely, diminishing returns from cooperative acts (*β* < 0) reduces the potential for cooperation to spread relative to the additive case.

The term *β*(⟦^2^*ρ*_*A*_⟧ − ⟦^2^*ρ*_*S*_⟧) is particularly interesting since it may be of the opposite sign to *P*_1_ and *P*_2_. ⟦^2^*ρ*_*A*_⟧ is the frequency variance, across the population, in the amount of cooperation experienced by each cooperator, while ⟦^2^*ρ*_*S*_⟧ is the corresponding variance in cooperation experienced by each non-cooperators. (⟦^2^*ρ*_*A*_⟧ − ⟦^2^*ρ*_*S*_⟧) > 0 when the variance in cooperation directed at cooperators is greater than that of cooperation directed at non-cooperators. Note that (⟦^2^*ρ*_*A*_⟧ − ⟦^2^*ρ*_*S*_⟧) is not necessarily zero, even when the “relatedness”-like term is zero ($P_{1}=({\overset{-}{\rho }}_{A}-{\overset{-}{\rho }}_{S})=0$) (Fig 3B).

If *β* > 0 and (⟦^2^*ρ*_*A*_⟧ − ⟦^2^*ρ*_*S*_⟧) > 0, or if both are negative, then cooperation can increase in frequency even if $({\overset{-}{\rho }}_{A}-{\overset{-}{\rho }}_{S})=0$ or slightly negative. Put another way: If the fitness effects of cooperation are compounding (*β* > 0), then cooperation can increase if the amount of cooperation directed at cooperators is more variable than that directed at non-cooperators (even if the mean values are the same). Note, we are separating the effects of fitness from the phenotype simply because we think the biological components contributing to the evolution of cooperation written in this way is more informative, not necessarily more intuitive. When *β* > 0, individuals gain an increasing benefit from the cooperation they receive. The cooperation an individual experiences and the benefits gained from those cooperative interactions are two biologically distinct factors. Similarly, cooperation can increase if *β* < 0 (diminishing returns from cooperation) and if the amount of cooperation experienced by cooperators is more predictable than that experienced by non-cooperators (⟦^2^*ρ*_*A*_⟧ − ⟦^2^*ρ*_*S*_⟧) < 0).

**Fig 4:**
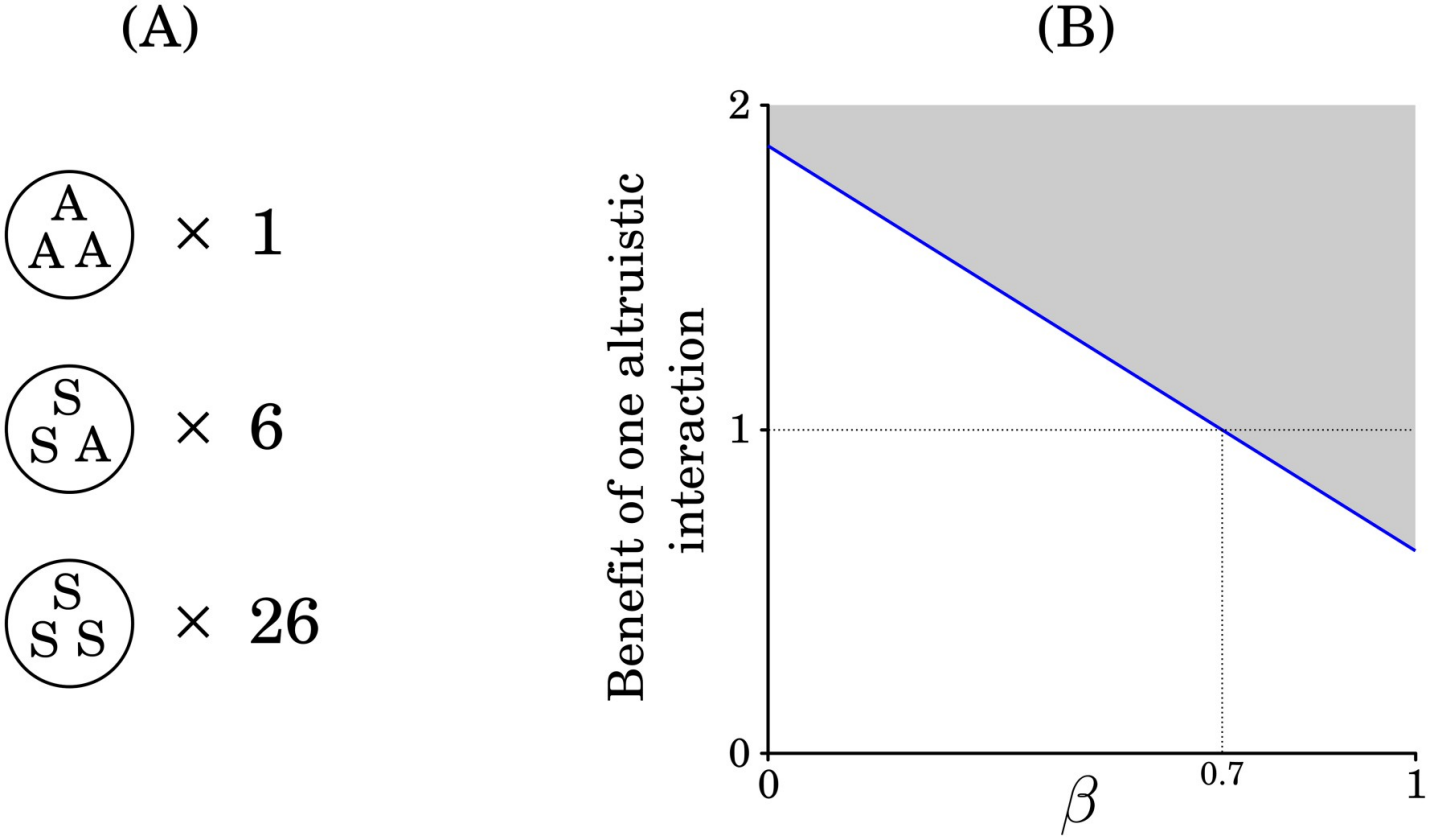
Example of the influence of non-additive benefits, *β*. (A) Diagram of a population of 99 individuals, 9 cooperators and 90 non-cooperators that interact in groups of 3. In this case, 1 group contains all cooperators, 6 groups contain one cooperator, and 26 groups have no cooperators. For this example, *P*_1_ = 0.533, *P*_*v*_ = 0.773, and *P*_2_ = 0.427. (B) Plot of the benefit to a recipient of one (and only one) cooperative interaction against the value of *β*. If *C* = 1, then cooperation can increase in frequency anywhere within the shaded region.

Non-additivity of benefits is likely to be relevant in cases in which cooperators are relatively rare, and occasionally grouped (dense groups are more common than expected by chance, but are still rare). In such cases, *P*_*v*_ will tend to be relatively large (*P*_2_ may be as well), making the value of *β* important. As an example, Fig 4 shows a hypothetical case in which in which a few cooperators are grouped, while most are dispersed among non-cooperators. In this case, *P*_1_ is positive (because of the grouped cooperators), but *P*_*v*_ is even larger. Non-additivity of benefits has a substantial effect in cases like this. In the additive case (*β* = 0), if the cost of cooperation is set at *C* = 1, cooperation could increase in frequency if the benefit of a single cooperative act (the benefit of interacting with one and only one cooperator) is greater than 1.875. As *β* increases, this threshold comes down. For *β* > 0.7, cooperation can increase in this case even if the benefit of a single cooperative interaction is less than the cost to the cooperator. Note though the average relative fitness benefits of a cooperative interaction is greater than the costs. The reason for this is that the rare group of all cooperators substantially increases the variance in *ρ*_*A*_.

If *P*_*v*_ and *β* are both positive, then there is the potential for cooperation to increase even if *P*_1_ and *P*_2_ are both negative. To see this, note that when *P*_*v*_ ≫ 0, some cooperators experience a lot of cooperation, while others experience very little. If *β* > 0, then the fitness benefits experienced by those with high *ρ*_*A*_ (experience much cooperation) will outweigh the reduced fitness of those who experience little cooperation (low *ρ*_*A*_). This is just a manifestation of Jensen’s inequality—resulting from the non-linearity of fitness effects [36,60].

Cooperation is also favored if *P*_*v*_ and *β* are both negative. Here, the individuals who experience the most cooperation are non-cooperators, but they gain relatively little due to the diminishing returns of cooperation (*β* < 0 line in Fig 2). In this case, the individuals experiencing the least cooperation are likely to also be non-cooperators (since their variance is relatively large), and these individuals do particularly poorly because of the abrupt decline in *B* at low values of *ρ*_*i*_.

Note that, so far, this is a deterministic model (each individual has a known fitness value), so the only variation in cooperation is captured by the variance across the population. The terms in Eq 7 are not yet random variables. In the next section, we allow fitness to be a random variable (each individual has a distribution of possible fitness values). In the stochastic case, another kind of fitness variance–probability variance—will arise that can have a very different effect on evolution.

### The non-additive, stochastic case

Eqs 3–7 treat fitness as a predictable determinate value; this assumes we can predict precisely how many descendants/offspring an individual will leave. In general, this is not possible. Introducing stochasticity means treating fitness as a random variable, which has a probability distribution of possible values. If individual fitness (*w*_*i*_) is a random variable, then so is mean population fitness ($\overset{-}{w}$) (Table 2). In this case, modeling evolution requires that we consider each individual’s relative fitness: $\Omega_{i}=\frac{w_{i}}{\overset{-}{w}}$(conditional on $\overset{-}{w}\neq 0$) [36,57,60,66,67]. The condition for cooperation to increase is now that the average expected relative fitness of cooperators is higher than that of non-cooperators. Using ^ (“hat”) for the expected value of a random variable, our condition can be written:

$${\hat{\Omega }}_{A}>{\hat{\Omega }}_{S}.$$ (8)

Eq 8 is the condition for a trait to be expected to increase under stochastic selection with constant heritabilities. The critical difference between using absolute fitness in Eq 6 and relative fitness in Eq 8 is that ${\hat{\Omega }}_{i}$ is the expected value of a ratio of random variables [67]. This means that ${\hat{\Omega }}_{i}$ is not just a function of the expected values of *w*_*i*_ and $\overset{-}{w}$ but also of the entire distributions of possible *w*_*i*_ and $\overset{-}{w}$ values. In particular, the probability variance in individual fitness (≪^2^*w*_*i*_≫) becomes an important factor in evolution [7,38,57]. This makes it possible to satisfy Eq 8 even when the mean absolute fitness of cooperators is lower than that of non-cooperators, meaning that Eq 6 is not satisfied. Directional evolutionary processes that result from $\overset{-}{w}$ being a random variable are called directional stochastic effects [57]. These effects can drive the evolution of cooperation in ways that are invisible to deterministic models.

We can rewrite Eq 8 by expanding ${\hat{\Omega }}_{i}$ in a Taylor series [57]. This yields the following condition for cooperation to increase (see Methods):

w-^A-w-^SH(w-)>∑k=1∞(-1)k⟪w-A-w-S,w-k⟫w-^k+1. (9)

Introducing stochasticity does not just ‘add noise’ to our result; instead, Eq 9 exposes new processes that influence the evolution of cooperation generally. On the left side of Eq 9, the difference between the expected absolute fitness of cooperators and that of non-cooperators is divided by the harmonic mean of $\overset{-}{w}$. Note, the right side of Eq 9 is not zero, as it would be in the deterministic case, but instead is a series containing the joint central moments of *w*_*i*_ and $\overset{-}{w}$, the same expansion, from Rice (2008), is also used in Kennedy et al. (2018).

As in the deterministic case, we can write our absolute fitness in terms of the benefits and cost of cooperation and the amount of cooperation experienced. However, *B*, *C*, and *ρ*_*i*_ are now random variables–meaning that they can covary with one another within an individual and across the population. Considering only the first order terms in the summation on the right hand side of Eq 9 and denoting the frequency of cooperators and non-cooperators by *f*_*A*_ and *f*_*S*_ respectively, the condition for cooperation to be expected to increase is now:

B^0P^1+⟪B0,P1⟫H(w-)+β^P^v+⟪β,Pv⟫H(w-)-β^P^2+⟪β,P2⟫H(w-)-C^H(w-)>fA⟪w-A2⟫w-^2-fS⟪w-S2⟫w-^2+(fS-fA)⟪w-A,w-S⟫w-^2. (10)

(Here, to illustrate the interaction of stochasticity and non-additivity, we only expand Eq 9 to the first central moment. This does not mean that higher order terms will not influence the evolution of cooperation. Discussion on expanding Eq 9 to higher order terms is found in the Methods section).

*P*_1_, *P*_*v*_ and *P*_2_ have the same basic interpretations that they had in the deterministic case, but they must be modified slightly now that absolute fitness and the amount of cooperation experienced are random variables:

- $P_{1}=({\overset{-}{\rho }}_{A}-{\overset{-}{\rho }}_{S})$ → The difference between the average degree to which cooperation is experienced by cooperators and the average degree to which it is experienced by non-cooperators.
- Pv=(⟦ρA2⟧+⟪ρA2⟫-)-(⟦ρS2⟧+⟪ρS2⟫-) → The difference between the total variance in cooperation experienced by cooperators (including the frequency variance in cooperation experienced across cooperators and the frequency average of the probability variance in cooperation experienced for each cooperator) and the total variance in cooperation experienced by non-cooperators. ⟪ρi2⟫- is simply the average across all individual’s distributions of ≪^2^*ρ*_*i*_≫. Now the total variance in cooperation experienced across and within for cooperators must be higher than for non-cooperators if this term is to be positive and aid the evolution of cooperation. In one scenario, this term could be positive even if on average cooperators have lower individual variance in cooperation experienced (⟪ρA2⟫-<⟪ρS2⟫-); as long as, the variance in cooperation experienced across cooperators is greater (⟦^2^*ρ*_*A*_⟧ > ⟦^2^*ρ*_*S*_⟧) and makes up the difference.

Group size does not show up as an explicit term, in Eq 10, but its effects are seen in *P*_*v*_. If group size is quite large, then each group is a representative sample of the population, meaning there is little variation across groups in cooperation experienced. Small groups matter because each ‘sample’ can vary greatly in cooperation experienced. Small groups thus increase that the likelihood some individuals experience cooperation but most do not. When those individuals are also cooperators, those groups do particularly well.

If individuals are randomly assorted, meaning that an individual’s distribution of cooperation experienced is binomial, then this probability variance increases as the group size decreases. Even if, on average, cooperators do not group together (⟦^2^*ρ*_*A*_⟧ < ⟦^2^*ρ*_*S*_⟧), *P*_*v*_ may still be positive if on average cooperators have the higher probability variance in cooperation experienced (⟪ρA2⟫->⟪ρS2⟫-). With small group sizes when cooperation is rare, few cooperators will experience cooperation, most will not.

- $P_{2}=({\overset{-}{\rho_{A}}}^{2}-{\overset{-}{\rho_{S}}}^{2})$ → The difference between the square of the average degree to which cooperation is directed towards cooperators and the analogous value for non-cooperators. This term will be influenced by non-additive benefits just as its deterministic counterpart.

In a completely deterministic case, these values collapse to the values given in Eq 7. Note, though, that the deterministic ‘*P’* functions are not simply the expected values of their stochastic counterparts.

We will discuss each of the terms in Eq 10 from left to right and discuss the equation as a whole in the Discussion. Note, all terms on the left side are divided by $H(\overset{-}{w})$ (the harmonic mean of mean absolute population fitness). This is important because, as the variance in mean absolute population fitness increases, holding the expected absolute fitness ($\hat{w}$) constant, the harmonic mean decreases, increasing the magnitude of the entire left hand side of Eq 10.

$+\frac{{\hat{B}}_{0}{\hat{P}}_{1}+⟪B_{0},P_{1}⟫}{H(\overset{-}{w})}:$ This term includes the product of the expected additive benefits (${\hat{B}}_{0}$), and the average expected degree to which cooperators interact with other cooperators verses non-cooperators (${\hat{P}}_{1}$) plus the probability covariance between the distributions of *B*_0_ and *P*_1_ (≪*B*_0_, *P*_1_≫). Note that probability operations capture variation in fitness within each individual and frequency operations capture variation across the population. ≪*B*_0_, *P*_1_≫ is the covariance between the distribution of possible additive benefits and the distribution of possible numbers of cooperative interactions. These terms are random variables because we cannot know with certainty how many benefits or interactions an individual will experience over its lifetime. In Eq 7, we assumed that there was zero variation in individual’s absolute fitness and so this covariance did not exist. If ${\hat{B}}_{0}{\hat{P}}_{1}>0$, this does not mean that ≪*B*_0_, *P*_1_≫ > 0, although this will make the evolution of cooperation more likely all else held equal.

$+\frac{\hat{\beta }{\hat{P}}_{v}+⟪\beta ,P_{v}⟫}{H(\overset{-}{w})}$: This term combines the degree of non-additivity in fitness effects (*β*) with the difference in the total variance in experienced cooperation. This term is the most unlike its deterministic counterpart. When we change deterministic terms *β* and *ρ*_*i*_ into distributions, we end up with the probability covariance between them and the expectations of those variables. The big difference is in the composition of *P*_*v*_. Now this includes the total variance in cooperation experienced (individual and population level) for both cooperators and non-cooperators. ≪*β*_0_, *P*_*v*_≫ is a probability covariance between two random variables; each individual has a joint distribution of *β* and *P*_*v*_. If ≪*β*, *P*_*v*_≫ >0, then the non-additive benefit effects are either accelerating or diminishing at the same time cooperators have higher total variance in cooperation experienced than non-cooperators ((⟦ρA2⟧+⟪ρA2⟫-)-(⟦ρS2⟧+⟪ρS2⟫-)>0). If ≪*β*, *P*_*v*_≫ < 0, this will make it harder for cooperation to increase, but if the expected values ($\hat{\beta }{\hat{P}}_{v}$) are positive and large the left side may still outweigh the right side of Eq 10.

$-\frac{\hat{\beta }{\hat{P}}_{2}+⟪\beta ,P_{2}⟫}{H(\overset{-}{w})}$: This term is subtracted from the left hand side of Eq 10, meaning all else held equal a positive value of ($\hat{\beta }{\hat{P}}_{2}+⟪\beta ,P_{2}⟫$) will reduce the potential for cooperation to evolve. This negative term may be surprising since all terms in the deterministic version (Eq 7), aside from the cost of helping, are positive. The probability covariance between the non-additive effects of cooperation and the degree in squared experienced cooperation directed towards cooperators (≪*β*, *P*_2_≫) appears because both *β* and *P*_2_ are random variables, without stochasticity ≪*β*, *P*_2_≫ = 0.

$-\frac{\hat{C}}{H(\overset{-}{w})}$: This term captures the cost of cooperation. In reality, there is no reason to assume the cost to cooperators be deterministic or additive. For simplicity of this discussion, *C* is a random variable but will we are not relaxing the assumption of non-additivity.

As noted above, all terms on the left hand side of Eq 10 are divided by the harmonic mean of mean absolute population fitness ($H(\overset{-}{w})$). Consequently, the absolute value of the left hand side of Eq 10 will tend to increase if $\overset{-}{w}$ becomes more unpredictable–as would be the case in a very small population or in a capricious environment. Whether this increases or decreases the potential for the evolution of cooperation depends on whether the sum of terms are positive or negative. If the sum is positive, then uncertainty in $\overset{-}{w}$ will increase the potential for the evolution of cooperation.

On the right hand side of Eq 9, the first order term contains a probability covariance between the difference in absolute fitness distributions of cooperators and non-cooperators and the first central moment of average absolute population fitness ($⟪w_{A}-w_{S},\overset{-}{w}⟫$). This term captures the relationship between the net benefits to cooperators and mean absolute population fitness ($\overset{-}{w}$). This covariance shows up in the derivation of ${\hat{\Omega }}_{i}$ because cooperative interactions between individuals cause their fitness distributions to be stochastically dependent (see Methods). We can break up the first order term on the right hand side of Eq 9 into three different terms, as shown in Eq 10:

+fA⟪w-A2⟫w-^2: This term is the product of the current frequency of cooperators in the population (*f*_*A*_) and the probability variance in mean absolute fitness for cooperators (⟪w-A2⟫). Note that ⟪w-A2⟫ is the variance in the distribution of possible mean absolute fitness values for cooperators; it is thus a property of an individual (unlike ⟦w^A2⟧– the frequency variance across the population in the expected absolute fitness of cooperators). If ${\overset{-}{w}}_{A}$ is not a random variable, this whole term will be zero (fA⟪w-A2⟫=0). In this case, there is no variation in cooperators’ absolute fitness. Therefore, this is no variation in mean absolute fitness (⟪w-A2⟫=0). This term can only be positive or zero; therefore, all else held equal, when cooperators are rare (*f*_*A*_ ≈ 0) or when the variance in mean absolute fitness for cooperators is small (⟪w-A2⟫≪1), the more likely the left hand side of Eq 10 is greater than the right, increasing the potential for cooperation to increase in frequency.

-fS⟪w-S2⟫w-^2: This term is the product of the frequency of non-cooperators in the population (*f*_*S*_) and the probability variance in mean absolute fitness for non-cooperators (⟪w-S2⟫). Because this term has a minus sign and *f*_*S*_ and ⟪w-S2⟫ are always non-negative, this entire term can only be negative or zero. Consequently, when fS⟪w-S2⟫≠0, this increases the potential for cooperation to increase in frequency, all else held equal.

$+\frac{{(f}_{S}-f_{A})⟪{\overset{-}{w}}_{A},{\overset{-}{w}}_{S}⟫}{{\hat{\overset{-}{w}}}^{2}}$: This term involves the difference in the frequency of non-cooperators and cooperators, multiplied by the probability covariance between their mean absolute fitness distributions. If cooperators are rare (*f*_*S*_ − *f*_*A*_ ≫ 0), then a highly negative probability covariance ($⟪{\overset{-}{w}}_{A},{\overset{-}{w}}_{S}⟫\ll 0$) will facilitate the evolution of cooperation, all else held equal.

### *In silico* experiments

To test some of the hypotheses generated from Eq 10, we set up two individual-based *in silico* experiments using Python. In order to illustrate the basic processes discussed above, we kept these simulations intentionally simple. This means we will make simplifying assumptions about which distributions to use and how individuals interact over one generation. Population size is relevant in terms of the directional stochastic effects and group size is relevant in terms of the variance in cooperation experienced. The simulations thus focus on relatively small populations of relatively small groups. We only aim to illustrate that the uncertainty in the level of cooperation matters and plays a key role in facilitating the evolution of cooperation when the population is structured into small groups.

Eq 10 can account for cooperative models that are considered weak or strong cooperation regimes. To elaborate this point, Simulation 1 is set up so that cooperators produce a diffuse product that is essentially a public good and thus benefits cooperators indirectly (but with that benefit being less than the cost, i.e. weak cooperation) [3,9,13,19,28,33,61]. Simulation 2 is set up similarly, but cooperators interact directly and do not benefit from their own behavior (i.e. strong cooperation) [8,9,12,13,28]. Table 3 is a quick reference of our individual-based simulation results. For more details on simulation design, see Methods section.

**Table 3:** Summarization of simulation results.

**----------Simulation 1 Results----------**						
			**Cooperators**	**Non-Cooperators**	$\hat{\overset{-}{P}}=-0.01$ ${\hat{\overset{-}{B}}}_{T}{\hat{\overset{-}{P}}}_{T}-{\hat{\overset{-}{C}}}_{T}=-0.25$ $\hat{\overset{-}{\Delta A}}=0.01$	
**Expected Relative Fitness** ($\hat{\Omega }$)			1.08	0.99		
**Expected Absolute Fitness** ($\hat{w}$)			2.39	15.28		
**Before interactions**	⟦w-^A2⟧		0.096			
	⟦w-^S2⟧			0.248		
	**⟦w-^2⟧pop**	0.061				
**After interactions**	⟦w-^A2⟧		0.493			
	⟦w-^S2⟧			1.082		
	⟦w-^2⟧pop	0.294				
**----------Simulation 2 Results----------**						
			**Cooperators**	**Non-Cooperators**	$\hat{\overset{-}{P}}=0.64$ ${\hat{\overset{-}{B}}}_{T}{\hat{\overset{-}{P}}}_{T}-{\hat{\overset{-}{C}}}_{T}=-0.21$ $\hat{\overset{-}{\Delta A}}=0.001$	
**Expected Relative Fitness** ($\hat{\Omega }$)			1.003	1.00		
**Expected Absolute Fitness** ($\hat{w}$)			6.16	10.26		
**Expected Benefits** ($\hat{B}$)			0.32	0.09		
**Population structure**			[SSSS]	[SSSS]	[SSAA]	[AAAA]
	⟦w-^2⟧group		0.25	0.25	0.225	0.191
**Before interactions**	⟦w-^A2⟧group				0.205	0.191
	⟦w-^S2⟧group		0.25	0.25	0.244	
	⟦w-^2⟧pop	0.06				
**After interactions**	⟦w-^A2⟧group				0.937	0.996
	⟦w-^S2⟧group		1.002	0.999	1.075	
	⟦w-^2⟧pop	0.251				

**Simulation 1:** To illustrate the consequences of non-additive benefits, we set the benefit function so the expected benefits of an cooperative act increase non-additively as the number of cooperators an individual interacts with increases ($\hat{\beta }=0.2$) (e.g. Fig 2 where *β* > 0). The cost is set so that an individual cooperator among all non-cooperators does not gain a benefit that outweights the personal cost of the cooperative phenotype ($\hat{C}=0.25$). The ratio of cooperators to non-cooperators in the population is set to 2:14 respectively. This simulation will show that cooperation can evolve even when cooperators are guaranteed to interact with non-cooperators each generation.

**Fig 5:**
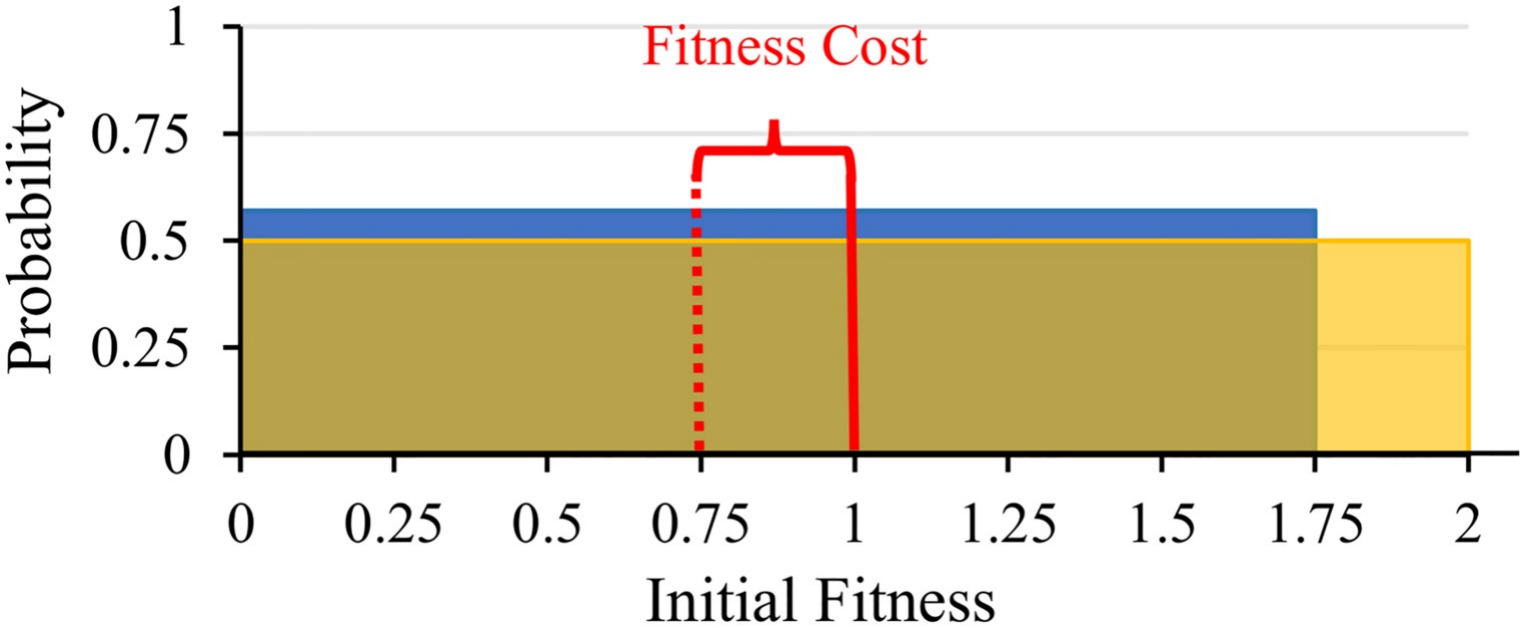
Baseline fitness disparity between phenotypes. Baseline probability absolute fitness distributions (*w*_0_) for cooperators (blue) and non-cooperators (yellow). Individuals are randomly assigned an expected absolute fitness from their respective distribution. The absolute fitness cost that cooperators pay for helping is the difference between the expected values of these distributions. This reduction in variance can, under certain environmental conditions, increase the magnitude of change in the frequency of cooperators.

From the population pool, four groups of four individuals each assemble at random. This is not a sample; this includes the entire population. The baseline absolute fitness distribution variance, mean and maximum for cooperators is reduced based on the inherent cost associated with the cooperative phenotype (Fig 5). Individuals are randomly assigned an expected baseline absolute fitness (${\hat{w}}_{0}$) from their phenotype-associated distribution before interactions occur. After group members interact and accrue potential benefits, final individual absolute fitness values are drawn from a Poisson distribution, the mean and variance of which is defined by the benefits and costs experienced by each individual.

We can see the effect of cooperation across cooperators, non-cooperators and the population. The frequency variance in expected mean absolute fitness for cooperators is lower than for non-cooperators before (⟦w-^A2⟧=0.096, ⟦w-^s2⟧=0.248) and after (⟦w-^A2⟧=0.493, ⟦w-^S2⟧=1.082) interactions within groups (Table 3). Though, both variances increase over the generation due to the effects of cooperation, non-cooperators increase much quicker. The frequency variance in expected mean absolute fitness across the population also increases over the generation (before interactions: ⟦w-^2⟧pop=0.061, after interactions: ⟦w-^2⟧pop=0.294), but its relatively low in respect to the final frequency variance in the expected mean absolute fitness of non-cooperators.

**Fig 6:**
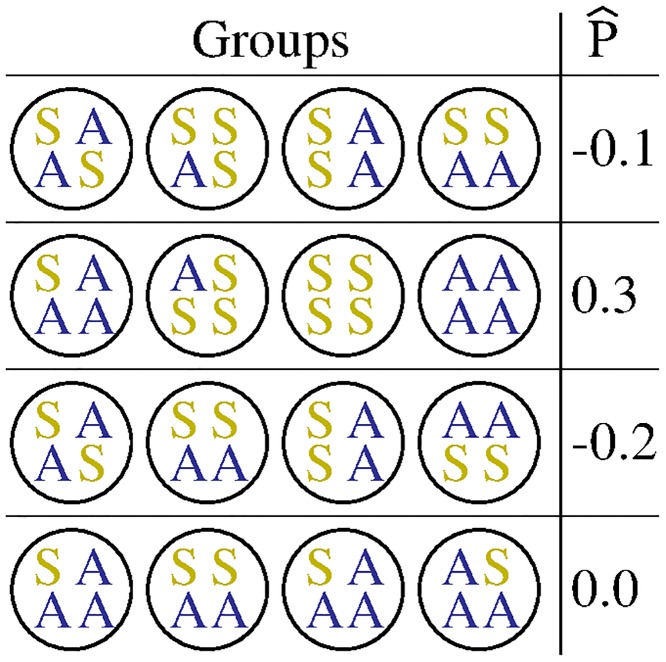
Possible interaction group arrangements. Four independent generations of possible populations consisting of four groups each. Each population produces a different $\hat{P}$ due to a random assortment of cooperators and non-cooperators. As it is more likely that cooperators encounter other cooperators, $\hat{P}$ increases.

In this simulation, $\hat{P}$ is different each generation for each group arrangement (Fig 6). On average, $\hat{\overset{-}{P}}=-0.01$, meaning that a non-cooperator is expected to experience more cooperative interactions than is a cooperator. Cooperators also experience lower expected absolute fitness than do non- cooperators (${\hat{w}}_{A}=2.39$, ${\hat{w}}_{S}=15.28$).

Because of non-additivity in benefits, though, cooperators experience higher expected benefits (${\hat{B}}_{A}=0.32$, ${\hat{B}}_{S}=0.09$) and increase in frequency after one generation (200,000 runs; $\hat{\overset{-}{\Delta A}}=0.01$; parental: *f*_*A*_ = 0.13 and offspring: *f*_*A*_ = 0.14), even though the total average expected cooperative effect is negative (${\hat{\overset{-}{B}}}_{T}{\hat{\overset{-}{P}}}_{T}-{\hat{\overset{-}{C}}}_{T}=-0.25$).

**Simulation 2:** Here, we show again that the expected absolute fitness alone is not a good predictor of the evolution of cooperation and highlight the importance of the variance in absolute fitness within groups. Initially, the population is arranged into four interaction groups consisting of four individuals each with assigned phenotypes (two groups consist of all non-cooperators, one group consists of all cooperators and one group is split with half non-cooperators and half cooperators). We set the expected cost of cooperation relatively high and the expected benefit so that it accrues in an additive fashion ($\hat{C}=0.25$, ${\hat{B}}_{0}=0.065$, $\hat{\beta }=0$). We are treating the benefits of cooperation as a single random variable, meaning that each individual experiences the same distribution of *B* values. This assumption is accurate only if the benefits of cooperation are additive, meaning that experiencing *z* cooperative acts leads to a total benefit of *zB*. With non-additivity, as in Simulation 1, the expected benefit ($\hat{B}$) per individual is a function of the number of cooperative acts experienced. Again, individual’s expected baseline absolute fitness and total expected number of descendants is drawn from a uniform (Fig 5) and Poisson distribution respectively.

Since we held group structure constant, we can look more closely at how the frequency variance in expected mean absolute fitness for cooperators and non-cooperators changes as cooperators join the group. This frequency variance decreases as the number of cooperators increases per group (Table 3). We can also look at the frequency variance in expected mean absolute fitness across cooperators and non-cooperators independently. The group containing two of each phenotype reveal that cooperators within a group of non-cooperator have lower variance in absolute fitness before (⟦w-^A2⟧mixedgroup=0.205, ⟦w-^S2⟧mixedgroup=0.244) and after (⟦w-^A2⟧mixedgroup=0.937, ⟦w-^S2⟧mixedgroup=1.075) interactions take place within the group. As in Simulation 1, the frequency variance in expected mean absolute fitness increases across the population over the generation (before interactions: ⟦w-^2⟧pop=0.06, after interactions: ⟦w-^2⟧pop=0.251).

In this simulation, due to the configuration of groups $\hat{\overset{-}{P}}=0.64$, cooperators are more likely than a non-cooperator to be the recipient of cooperation. Despite this, the total fitness effect is negative for cooperation because the cost heavily outweighs the benefits of helping (${\hat{\overset{-}{B}}}_{T}{\hat{\overset{-}{P}}}_{T}-{\hat{\overset{-}{C}}}_{T}=-0.21$). Due to this high cost, non-cooperators end up with the higher expected absolute fitness (${\hat{w}}_{S}=10.26$, ${\hat{w}}_{A}=6.16$). We would expect, with this information, cooperation to decline under a deterministic model (Eq 4). In fact, because cooperators have a lower average variance in absolute fitness and higher relative fitness in this case, cooperation increases on average (200,000 runs; $\hat{\overset{-}{\Delta A}}=0.001$; parental: *f*_*A*_ = 0.375 and offspring: *f*_*A*_ = 0.376).

Higher order moments are often not used due to the assumption their effects on the overall outcome are negligible as the population size increases. We only went out to the first order term because it results in the simplest form containing the components of interest: stochasticity, non-additivity, variances and covariances. Higher order moments (including skewness and kurtosis) can be included by using more of the terms on the right side of Eq 9 (see Methods). The effects of population size appear in the numerator and denominator of both sides of Eq 10 (also see Methods for Eqs 12 and 13). It is often assumed that these will have little effect in populations that are not tiny, but this is not necessarily true. The effects of higher moments drop off with increasing population size only when individual fitness values are stochastically independent. When individual fitness variation is correlated, as when much individual variation is due to variation in a shared environment, then higher moments may be important even in large populations [57].

Note that focusing on an inequality limits the result to yes or no, cooperation will increase or not. Our simulations were designed to test this binary outcome, and identify processes through which cooperation can increase when rare in a small population. We are also not evaluating the equilibrium level of cooperation attained, only whether it can increase when rare. Note, however, that because we are using relatively small populations, in all our simulations cooperation is maintained at least at a moderate frequency (>13%). If the left side of Eq 10 is negative and the right side is infinitesimally more negative, cooperation is expected to increase. In fact, if we increase the population size in Simulation 1 from 16 to 200 (with groups of 4 or 8) or 4000 (with groups of 4), the average expected change in cooperation is positive in all three cases respectively ($\hat{\overset{-}{\Delta A}}=0.00082295,0.0078839,3.7055E^{-5}$). We also saw similar results for Simulation 1. We should expect to see this drop off in the expected average change in cooperation because we are only including the effects of demographic stochasticity. We are assuming each individual experiences a different environment, which essentially gets incorporated into individual stochasticity in which individuals are stochastically independent. Therefore, we would expect a diminishing magnitude of the higher order terms as the population size increases (see Methods). If the environmental conditions affect individuals across the population, then this expectation does not necessarily hold as N gets large [57].

## Discussion

Cooperation can clearly evolve under various circumstances. The model presented here encompasses the well-established mechanisms of kin selection, reciprocal cooperation, and green beard effects, while also identifying other mechanisms involving non-additive fitness effects and stochastic selection.

### Non-additive benefits of cooperation

It is well supported that non-additive fitness benefits influence the evolution of cooperation [13,19,23,39,42–44,47–53,58]. Allowing variation in how the benefits of helping accrue as a function of group structure can dramatically change the probability of cooperation increasing. We will describe two possible non-additive cases that help to clarify the different conditions under which cooperation can arise, and argue that different conditions may often facilitate the evolution of cooperation in microorganisms relative to behaviorally complex animals.

If we ignore stochasticity and focus solely on non-additivity in benefits, Eq 7 is determined by the second (*βP*_*v*_) and third (*βP*_2_) term on the left side. This non-additivity creates new opportunities for the evolution of cooperation, relative to a deterministic, additive model where these terms would be zero. Eq 7 shows that an accelerating fitness function (*β* > 0) could facilitate the evolution of cooperation among non-relatives when cooperators are clumped by chance. In particular, Eq 7 shows that even if cooperators interact more often on average with non-cooperators than with other cooperators (*P*_1_ < 0), cooperation can still increase as long as cooperators have higher variance in their degree to which they encounter other cooperators (⟦^2^*ρ*_*A*_⟧ > ⟦^2^*ρ*_*S*_⟧) (Fig 2B). This means most cooperators do not interact with another cooperator but some do, and in those instances they do really well relative to other groups because of the non-additive fitness benefits. We might expect this sort of benefit function for a growing biofilm because the diffuse benefits of cooperation are not realized until a sufficient number of individual cells are producing beneficial compounds [6,21,45,64,68–70]. As the biofilm matures, the dynamics of Eq 7 will change. As cells produce offspring, they will likely increase their cooperation experienced (*P*_1_ > 0), but as the biofilm deteriorates the benefits of helping may diminish (*β* < 0; e.g. overcrowding). Cooperation can be facilitated nonetheless through the difference in their variance in cooperation experienced (⟦^2^*ρ*_*A*_⟧ < ⟦^2^*ρ*_*S*_⟧). The dynamics of the biological system change over time and what may contribute to the increase of cooperation may also change.

For the evolution of cooperation, decelerating benefit cases seem unlikely and even if they do happen, it must only be temporary. In fact, diminishing benefit cases (e.g. *β* < 0 in Fig 2) are quite common in examples like ‘helpers at the nest’, where one or two cooperators greatly help, but adding more cooperators correlates with diminishing returns. This has been demonstrated in a number of studies on species such as *Philetairus socius* [42,54], *Buteo galapagoens* [43], *Marmota flaviventri* [39], *Lamprotornis superbus* [52] and *Picoides borealis* [71]. Cooperation still increases in these cases because interactions are limited to close relatives or individuals that can remember past interactions, which both increase *P*_1_ and *P*_2_. The only way cooperation persists in these examples under a deterministic, non-additive model is due to (⟦^2^*ρ*_*A*_⟧ < ⟦^2^*ρ*_*S*_⟧).

In Simulation 1, individuals were randomly assorted (2 cooperators and 14 non-cooperators) into four groups. There are only three types of groups that can be produced: all non-cooperators, one cooperator or two cooperators. There are only two population combinations of these three groups within one generation (one possible run of the simulation): one cooperator in two groups (pop1) or two cooperators in one group (pop2). If we consider the parameters of Simulation 1 but set the initial population structure similar to pop1, then Eq 7 would not predict pop1 to produce more cooperators in the next generation (*β* > 0 and *P*_2_ and *P*_*v*_ < 0). If we consider pop2 in the same regard, Eq 7 would predict the evolution of cooperation (*β* > 0, *P*_2_ > 0 and *P*_*v*_ < 0). This is due to average positive assortment across the population, not the variance. Simulation 1 was ran 200,000 times and each generation could look like pop1 or pop2, but on average *P*_1_ and *P*_2_ < 0. With *P*_*v*_ < 0 in either case (pop1 and pop2), the addition of non-additivity in benefits could not solely predict the parameters of Simulation 1 would facilitate the evolution of cooperation.

The processes for the evolution of cooperation shown in Eq 7 require that cooperators sometimes encounter one another, meaning that there must be a few cooperators to begin with. In the next section, we will encounter a mechanism that can cause cooperators to increase in frequency even if they are initially very rare.

### Directional consequences of stochasticity

Expanding fitness in Eq 7 with stochasticity adds several new terms to the left side and completely transforms the right hand side from zero to a probability covariance between the difference in mean absolute fitness of cooperators and non-cooperators and mean absolute population fitness ($\frac{⟪{\overset{-}{w}}_{A}-{\overset{-}{w}}_{S},\overset{-}{w}⟫}{{\hat{\overset{-}{w}}}^{2}}$). Anything that reduces the value of this term will, all else held equal, increase the potential for cooperation to evolve. If the left hand side of Eq 9 is negative, there is the potential for cooperation to increase as long as this term is even more negative.

Denoting the frequencies of cooperators and non-cooperators as *f*_*A*_ and *f*_*S*_, respectively, we can expand $\frac{⟪{\overset{-}{w}}_{A}-{\overset{-}{w}}_{S},\overset{-}{w}⟫}{{\hat{\overset{-}{w}}}^{2}}$ to fA⟪w-A2⟫-fS⟪w-S2⟫+(fS-fA)⟪w-A,w-S⟫w-^2. Eqs 9 and 10 are equivalent (though Eq 10, as written, includes only the first order terms from Eq 9); they illustrate how different biological processes can facilitate the evolution of cooperation. Independent conditions under which the right side of Eqs 9 and 10 can be negative include: 1) the case in which cooperators have a lower variance in absolute fitness than do non-cooperators (⟪w-A2⟫<⟪w-S2⟫), 2) the case in which cooperation does relatively well when the population is declining ($⟪{\overset{-}{w}}_{A}-{\overset{-}{w}}_{S},\overset{-}{w}⟫<0$), and 3) the case in which cooperators and non-cooperators have (partially) independent fitness distributions when cooperation is rare (${(f}_{S}-f_{A})⟪{\overset{-}{w}}_{A},{\overset{-}{w}}_{S}⟫<0$).

All three of these processes can be classified as directional stochastic effects [57]. These are evolutionary forces that yield directional change (unlike drift), but that exist only when there is stochastic variation in individual absolute fitness (*w*_*i*_) and in mean absolute population fitness ($\overset{-}{w}$). We discuss these cases in order.

#### Cooperators have lower variance in fitness

This effect was apparent in both Simulations 1 and 2, where $\hat{\overset{-}{P}}<0$ and $\hat{C}>>{\hat{B}}_{0}$, respectively. There, the cost of cooperation manifests as a reduction in the maximum absolute baseline fitness (exaggerated in Simulation 2). This reduces both the expected absolute fitness of cooperators and their variance (Fig 5). Possession of the cooperative trait inherently reduces the variance in the distribution of an cooperator’s absolute fitness. In certain contexts, this could facilitate the evolution of cooperation and in others it may inhibit it.

In Simulation 1 and 2, the benefits cooperators gain through their interactions with other cooperators increases their expected absolute fitness but also their variance in mean absolute fitness, although both were still lower than for non-cooperators. As the frequency of cooperators increases, the less flexibility cooperators have in their mean absolute fitness variance and remain smaller than the corresponding non-cooperators term (fA⟪w-A2⟫<fS⟪w-S2⟫). Cooperators in Simulation 1 were able to increase in frequency over one generation because they were rare and had lower variance in mean absolute fitness (fA⟪w-A2⟫), despite the expected terms on the left side of Eq 10 being zero or negative. These effects may have helped cooperators in Simulation 2 as well, although they also gained benefits from the left side of Eq 10 (${\hat{B}}_{0}{\hat{P}}_{1}>0$).

Throughout this discussion and in Simulations 1 and 2, we assumed the benefits of helping and the ‘*P*’ terms do not covary. We have only done this for simplicity of this discussion, but in reality these probability covariances could be very influential. In fact, these covariances may have the opposite sign to their expected product counterparts and are of interest for further study.

In general, this effect of reduced variance in absolute fitness is likely to be important if cooperators are grouped, and the cooperative act itself buffers individuals against environmental fluctuations. One example includes the production of compounds that add mechanical strength to a biofilm. Some biofilm-forming bacteria create coaggregation bridges that bring non-coaggregation species into closer proximity. These bridges are vital for neighboring communication and overall biofilm integrity (i.e. insurance hypothesis, stability-diversity hypothesis, social insulation, etc.) [31,48,64,72]. If we consider the formation of these bridges an cooperative act, then this facilitates the evolution of cooperation because it also happens to group cooperators. In such a case, groups with cooperators have lower variance in their absolute fitness distribution relative to non-cooperators (as we saw in Simulation 2). When cooperators increase in frequency it is with greater magnitude than when they decrease in frequency. The consequence is that the expected average change in the frequency of cooperators is positive. This is an example of a directional stochastic effect [57].

Reduction in cooperators’ absolute fitness variance is related to ‘cooperative bet-hedging’ as discussed by Kennedy, Higginson, Radford and Sumner (2018). They define this strategy as one that facilitates the evolution of cooperation by reducing the variance in the fitness distributions of the cooperator’s relatives, even if the average fitness of those relatives is reduced. In accordance, Eq 10 (Eq 9 expanded to the first central moment) shows that if cooperators reduce their variance in absolute fitness (⟪w-A2⟫) relative to non-cooperators (⟪w-S2⟫), all else held equal cooperation is likely to increase. They highlight the importance of stochasticity in fitness and detail evolutionary predictions that would otherwise be overlooked under a deterministic Hamilton’s rule. Directional stochastic effects, while influenced by the probability variance in absolute fitness, can lead to the increase of cooperation even when cooperators have a lower mean fitness [57].

Another empirical example in which cooperation influences absolute fitness variance is found in superb starlings (*Lamprotornis superbus*). Rubenstein (2011) shows that as group size (direct relation to number of helpers) increases, group mean absolute fitness increases and group variance in absolute fitness decreases. This cooperative buffering or ‘insurance’ provided by grouping reduces the fitness variance of participating individuals and has a significant effect when there is environmental instability [73,74]. It is likely that *P*_1_ will be positive, since helpers are usually related within groups. Eq 10 shows that as long as cooperators do not greatly outnumber non-cooperators (*f*_*A*_ < *f*_*S*_), the decrease in variance in absolute fitness due to grouping can drive the evolution of cooperation.

#### Cooperators do well (relative to non-cooperators) when the population is declining

This is the most direct interpretation of Eq 9. If the value of (${\overset{-}{w}}_{A}-{\overset{-}{w}}_{S}$) is largest when $\overset{-}{w}$ is small, then the right side of Eq 9 will be negative. Note, for cooperation to evolve it need not be the case that the mean absolute fitness of cooperators is ever greater than that of non-cooperators as long as this probability covariance is negative ($⟪{\overset{-}{w}}_{A}-{\overset{-}{w}}_{S},\overset{-}{w}⟫$).

This condition will hold if, for example, cooperators tend to group and the benefits of cooperation are most pronounced in harsh environmental conditions [7]. This has been described in the social weaver, *Philetairus socius*. In these birds, adverse breeding conditions, such as low rainfall or large colony size, increases the demand for helpers [42]. As poor conditions continue, the per capita growth rate continues to decline, $\overset{-}{w}$. Nonetheless, cooperation still increases in frequency (*f*_*A*_ > 0). This combination will eventually make the right side of Eq 10 less negative, thus reducing the potential for an increase in cooperation. Cooperation continues to evolve as long as cooperators have lower variance in absolute fitness (⟪w-A2⟫) than non-cooperators and increasingly higher mean absolute fitness (${\overset{-}{w}}_{A}-{\overset{-}{w}}_{S}$), all else held equal.

Cooperation increases in species like the white-winged choughs (*Corcorax melanorhamphos*) due to a ‘buffering effect’ (similar to what is seen in the superb starlings, and non-additive absolute fitness benefits) [40]. Collaborative benefits come in the form of young care and group defense. Group sizes range from about 3 to 20 individuals, though the benefits from helping taper off around 15 individuals. In this case, $⟪{\overset{-}{w}}_{A}-{\overset{-}{w}}_{S},\overset{-}{w}⟫$ will be negative when unpredictability in their environment (such as an increase in the variance of predator visits) increases (decreasing $\overset{-}{w}$) as groups of cooperators increase, in turn increasing ${\overset{-}{w}}_{A}$ relative to ${\overset{-}{w}}_{S}$.

Cooperative behavior that is conditional on population dynamics (the magnitude of $\overset{-}{w}$) is also seen in biofilms. Xavier, Kim and Foster (2011) found no difference between the average fitness of cooperators and non-cooperators; nonetheless, cooperators increase due to ‘metabolic prudence’. Groups of *Pseudomonas aeruginosa* cooperators secrete biosurfactants and relocate by swarming. To avoid helping non-cooperators, cooperators only initiate biosurfactant synthesis when $\overset{-}{w}$ is decreasing [75]. In this case, ‘metabolic prudence’ increases the likelihood that cooperators interact with other cooperators (*P*_1_) and decreases the variance in absolute fitness of cooperators (⟪w-A2⟫) by producing secretions when the population growth rate slows ($⟪{\overset{-}{w}}_{A}-{\overset{-}{w}}_{S},\overset{-}{w}⟫<0$). This mechanism increases the left side and decreases the right side of Eq 10, facilitating the evolution of cooperation.

#### Cooperators are rare and exhibit independent fitness variation

This is related to the concept of induced overdominance, which has long been recognized in the population genetics literature but is rarely invoked in discussions of cooperation [7,57,76,77]. In this case, an individual’s relative fitness (*Ω*_*i*_) is a distribution with the expected value, variance, and higher moments all contributing to evolution. (Eq 15 –see Methods) [7,37,57]. Consider, for purposes of illustration, a case in which the relative and absolute fitness variation of cooperators is completely independent of the fitness variation of non-cooperators so that $⟪{\overset{-}{w}}_{A},{\overset{-}{w}}_{S}⟫=0$, and the variances of each are equal (⟪w-A2⟫=⟪w-S2⟫). In this case, the right side of Eq 10 will be negative whenever the frequency of cooperators is lower than the frequency of non-cooperators (*f*_*A*_ < *f*_*S*_). This hypothetical example is an extreme case; the fitness distributions of different strategies need only be partially independent, and the variances need not be the same. We expect that the fitness of cooperators will vary at least somewhat independently of that of non-cooperators because a component of their fitness, the cost of helping, is not experienced by non-cooperators.

This process, in which a strategy can increase simply because the fitness of individuals that express it are stochastically independent of the rest of the population, is a very general phenomenon that is not specific to cooperation. Its relevance here is that it is strongest when the variant strategy is rare (and disappears when all strategies are at equal frequencies). It thus provides a mechanism, emerging naturally from our model that can cause cooperation to initially increase to an intermediate frequency [1,21,26,30]. This is significant because most mechanisms for the evolution of cooperation require that there be enough cooperators in the population in which they can interact [8,10,24,25,28,45,78].

Though there are many routes to the evolution of cooperation, they all fall under the same general condition given in Eq 10. This model allows us to elaborate on established mechanisms, such as kin selection and reciprocal cooperation, while also revealing other processes—involving non-additive and stochastic fitness effects—through which helping behavior can increase. We have focused our discussion on examples of cooperation but this model can extend to other types of cooperation. Fitness can be partitioned in different ways to address specific details of the biological system under study. With knowledge of a particular system, assumptions can be made about which benefit and cost functions are more realistic and if they should change over time as the dynamics change within the population or group.

In real populations, fitness is always stochastic (we will never know ahead of time *exactly* how many descendants an individual will leave), and the benefits of many kinds of helping behaviors are likely to be non-additive. We saw through mathematical modeling and individual-based simulations that the evolution of cooperation can be facilitated when cooperators have lower variance in individual absolute fitness, when cooperators form small groups or clusters (relative to the whole population) when the environment is unpredictable and when cooperators are rare and somewhat vary independently from non-cooperators among other potential evolutionary pathways. We thus expect that the mechanisms presented here are acting in many natural populations and may contribute substantially to the prevalence of the helping behavior.

## Methods

### Derivation of non-additive, stochastic model

A general model should deduce any situation, and that means we must allow the benefits to accrue additively or non-additively (e.g. accelerating, diminishing, sigmodal, any non-linear function). Therefore, the benefits of cooperation must have a tunable slope. Eq 5 generates a functional relationship between *B* and ${\overset{-}{\rho }}_{i}$ that can change over time. If we combine Eqs 5 and 6, then the mean absolute fitness for cooperators and non-cooperators is respectively:

$${\overset{-}{w}}_{0}+B_{0}{\overset{-}{\rho }}_{A}+\beta \overset{-}{\rho_{A}^{2}}-\overset{-}{C}>{\overset{-}{w}}_{0}+B_{0}{\overset{-}{\rho }}_{S}+\beta \overset{-}{\rho_{S}^{2}}.$$ (11)

For simplicity, we are assuming ${\overset{-}{w}}_{0}$ is the same for cooperators and non-cooperators. This is not necessary true and should not be overlooked. Now move terms across the inequality and cancel the baseline absolute fitness. We get Eq 7 from Eq 11 by expanding the $\overset{-}{\rho_{i}^{2}}$ terms using the rule: $\overset{-}{a^{2}}=⟦a^{2}⟧+{\overset{-}{a}}^{2}$.

All terms in Eq 11 are constants, meaning we are assigning exact numbers to fitness components that cannot be precisely determined from information of the current state. We want to allow stochasticity, or uncertainty, and treat each term as a random variable. We must now use relative fitness (*Ωi*), instead of absolute fitness (*w*_*i*_), because both *w*_*i*_ and $\overset{-}{w}$ are random variables [57,66]. We use ≪^*k*^ *w*≫ to denote the *k*th central moment of individual absolute fitness, *w*_*i*_. This Taylor expansion allows us to approximate each individual’s relative fitness function. The expected relative fitness of an individual can be written as:

Ω^i=w^iH(w-)-⟪wi,w-k⟫w-^i+1+⋯≡w^iH(w-)+∑k=1∞(-1)k⟪wi,w-k⟫w-^k+1, (12)

where ⟪wi,w-k⟫=E[(wi-w^i)(w--w-^)k] [57].

If we expand ⟪wi,w-k⟫ for *k* = 1, then ⟪wi,w-⟫=⟪wi,1N∑j=1Nwj⟫, where *N* is population size and *w*_*j*_ is the absolute fitness of individuals other than individual *i*. We can pull out the constant and expand $\frac{1}{N}\sum_{j=1}^{N}w_{j}$ further so that $\frac{1}{N}\sum_{j=1}^{N}w_{j}=\frac{1}{N}⟪w_{i},w_{i}⟫+\frac{1}{N}\sum_{j\neq i}⟪w_{i},w_{j}⟫$. We get a probability variance of individual *i* (≪*w*_*i*_, *w*_*i*_≫) and a probability covariance ((≪*w*_*i*_, *w*_*j*_≫) between individual *i* and all other individuals but excludes the covariance between individual *i* and itself [36,57]. We end up with two terms that describe when individuals are stochastically independent or dependent: ⟪wi,w-⟫=1N⟪wi2⟫+N-1N⟪wi,wj≠i⟫-. We can put this expansion back into Eq 12 and see the effects of the first raw moment ($\frac{{\hat{w}}_{i}}{H(\overset{-}{w})}$) and the first central moment (-⟪wi2⟫Nw-^2-(N-1)⟪wi,wj≠i⟫-Nw-^2) of their distribution on relative fitness of individual *i*:

Ω^i=w^iH(w-)-⟪wi2⟫Nw-^2-(N-1)⟪wi,wj≠i⟫-Nw-^2+∑k=2∞(-1)k⟪wi,w-k⟫w-^k+1. (13)

$\frac{(N-1)\overset{-}{⟪w_{i},w_{j\neq i}⟫}\;}{N{\hat{\overset{-}{w}}}^{2}}$ will be set to zero if individuals are stochastically independent. If individuals are stochastically dependent (and likely in cases of cooperation), then random factors that affect their fitness also affect the fitness of others; therefore, $\frac{(N-1)\overset{-}{⟪w_{i},w_{j\neq i}⟫}\;}{N{\hat{\overset{-}{w}}}^{2}}\neq 0$ [57].

Stochasticity can be added to Eq 7 by using Eq 13 for cooperators and non-cooperators relative fitness and using these rules: $\hat{ab}=\hat{a}\hat{b}+⟪a,b⟫$, ⟦a2⟧^=⟦a^2⟧+⟪a2⟫--⟪a-2⟫, and a-2^=⟪a-2⟫-a-^2:

B^0(ρ-^A-ρ-^S)+⟪B0,(ρ-A-ρ-S)⟫+β^((⟦ρ^A2⟧+⟪ρA2⟫--ρA-^2)-(⟦ρ^S2⟧+⟪ρS2⟫--ρS-^2))+⟪β,(⟦ρA2⟧-⟦ρS2⟧)⟫-β^(ρA-^2-ρS-^2)+⟪β,(ρA-2-ρS-2)⟫-C^>0. (14)

So far, we have only expanded terms on the left side of Eq 9. If we expand w- on the right side so that *k* = 1, then w-=1N(∑i=1nAwA+∑i=1nSwS)=fAw-A+fSw-S, where *n*_*A*_ is the total number of cooperators and *n*_*S*_ is the total number of non-cooperators in the population. We end up with four new terms, two of which are probability variances and the others collapse into a single probability covariance: ⟪w-A-w-S,w-⟫=fA⟪w-A2⟫-fS⟪w-S2⟫+(fS-fA)⟪w-A,w-S⟫. If we plug this set of terms into the right side of Eq 14, we get Eq 10. We can rewrite Eq 10 into a condensed form:

B^0P^1+⟪B0,P1⟫+β^P^v+⟪β,Pv⟫-β^P^2+⟪β,P2⟫-C^H(w-)>⟪w-A-w-S,w-⟫w-^2. (15)

We could easily expand the right side of Eq 15 to higher central moments (*k* = 2, 3 etc.). All else held equal, the *k* = 2 term (⟪w-A-w-S,w-2⟫w-^3) will be negative and potentially beneficial to the evolution of cooperation if it were a large, positive probability covariance [37,57]. The *k* = 3 term (⟪w-A-w-S,w-3⟫w-^4) will be positive, potentially boosting cooperation if a highly negative covariance.

### *In silico* experiments

**Simulation 1:** Each parental generation (non-overlapping) or each run of the simulation, the population ratio is set to 2:14 for the number of cooperators and non-cooperators, respectively. As mentioned earlier, *ρ*_*i*_ will be calculated differently dependent on the context of the biological system under study. Group size (G) may vary across the population; therefore, we must count each cooperative encounter and not divide by G-1 as in Simulation 2 (Table 4). In this case, we calculate cooperation experienced by an cooperator or a non-cooperator in group *g* as $\rho_{A_{g}}=n_{A_{g}}-1$ and $\rho_{S_{g}}=n_{A_{g}}$, respectively. Now we can calculate the average amount of cooperation experienced by cooperators and non-cooperators, respectively: ${\overset{-}{\rho }}_{A}=\frac{\sum_{g}n_{A_{g}}*\rho_{A_{g}}}{N_{A}},$, ${\overset{-}{\rho }}_{S}=\frac{\sum_{g}n_{S_{g}}*\rho_{S_{g}}}{N_{S}}$.

**Table 4:** Term and symbol definitions for Simulation 1 & 2.

G	number of individuals in a group; group size
$n_{A_{g}}$	number of cooperators in group *g*
$n_{S_{g}}$	number of non-cooperators in group *g*
***N*_*A*_**	total number of cooperators in the population
***N*_*S*_**	total number of non-cooperators in the population
$\rho_{A_{g}}$	cooperation experienced by an cooperator in group *g*
$\rho_{S_{g}}$	cooperation experienced by a non-cooperator in group g
${\overset{-}{\rho }}_{A}$	average amount of cooperation experienced by cooperators
${\overset{-}{\rho }}_{S}$	average amount of cooperation experienced by non-cooperators

In this simulation, individuals (haploid, asexual) are randomly assigned (equal chance) to one of the four interaction groups (constant group size of four) each generation. Although mathematically we are allowing for variation in group size, we want to make consistent comparisons across groups that differ in their numbers of cooperators. Setting the number and size of groups will make it more difficult for cooperation to increase in frequency because cooperators will always end up in a group with some non-cooperators. When group size is variable it is possible in some generations that the two cooperators will group alone (i.e. positive assortment–whether it be due to relatedness, trait recognition or just by chance). In this instance, the benefits of cooperation are directed only at cooperators, making it easier for cooperation to evolve. In general, as group size decreases, the variation between groups increases. This is something we want to highlight through the ‘*P*’ terms in Eq 10.

Random colonization of groups means that $\hat{P}$ will be different for each group, each generation because the arrangement of cooperators will change. The benefits of cooperation are a function of the cooperative environment experienced. In this simulation, as the number of cooperators increase per group, the benefit increases non-additively. This does not have to be the case. The benefit function may take many forms, depending on the biological system, including variants of the functions shown in Fig 2. In a realistic case, the benefits will not continue to increase exponentially as the number of cooperative interactions increase. Instead, the function may taper off after some number of interactions, approaching a sigmodal shape. In this simulation, we are only looking over one generation and the number of parental cooperators is always two.

We can minimize the number of cooperators per group by allowing cooperators to benefit from their own diffusion of products. If a cooperator is in a group with all non-cooperators, the benefit it gains from its own diffusion is less than the cost paid for being a cooperator. The benefits of cooperation to each cooperator surpasses the individual cost only when both cooperators end up in the same group. This is similar to what some describe as ‘synergistic effects’ [17,18,22]; although, we are considering these effects at the individual level instead of the group level. This ties into the earlier discussion on non-additivity.

For simplicity, we only consider the expected cost, $\hat{C}$, and assume that *B* and *P* do not covary. We assume that a cooperator’s cost is not a function of how many interactions an individual has; instead, the cost to a cooperator is inherit; this phenotype confers a fitness disadvantage from the very beginning. Individuals start the simulation with a randomly chosen baseline absolute fitness drawn from a uniform distribution (Fig 5). With more information about a particular biological system, these distributions can be tuned to better represent what the actual distribution might be. After interactions within groups, the sum of each individual’s baseline fitness and benefits gained by interacting with cooperators determines the mean and variance of their final fitness distribution (Poisson distribution). This is the number of offspring that a particular individual will produce over its lifetime (one generation). This additional random step (Poisson distribution) takes into account random variation other than interactions with neighboring individuals (e.g. environmental, reproduction).

**Simulation 2:** We set the population each generation (non-overlapping) by assuming group size constant (4 groups of 4 haploid, asexual individuals each) within the simulation and mathematically. With this assumption, we can scale individual’s cooperation experienced for cooperators and non-cooperators, so that the maximum is 1: $\rho_{A_{g}}=\frac{n_{A_{g}}-1}{G-1}$ and $\rho_{S_{g}}=\frac{n_{A_{g}}}{G-1}$, respectively (Table 4). To calculate the average cooperation experienced for cooperators and non-cooperators, plug these terms into the ${\overset{-}{\rho }}_{A}$ and ${\overset{-}{\rho }}_{S}$ equations written out above for Simulation 1.

In order to magnify the role of variance and expose its influence on the evolutionary outcome of these groups, we will also set an exact configuration of individuals described in the Results. Again, $\hat{C}$ is a fixed value and *B* and *P* do not covary. In this case, cooperators do not benefit from their own diffusion products. The interaction between individuals of a group can be thought of in two ways: 1) each individual has three independent one-on-one interactions over the generation (e.g. single play game strategies) or 2) each individual has one interaction with all three individuals at the same time (e.g. swarming). We are assuming the benefits gained from each of these scenarios is the same, though, in reality they could be different.

We assume that an individual’s fitness is determined as in Simulation 1 –baseline absolute fitness is randomly chosen from a uniform distribution, interactions between individuals take place within groups and the total expected number of offspring is randomly selected from a Poisson fitness distribution.
